# P7C3 inhibits GSK3*β* activation to protect dopaminergic neurons against neurotoxin-induced cell death *in vitro* and *in vivo*

**DOI:** 10.1038/cddis.2017.250

**Published:** 2017-06-01

**Authors:** Chao Gu, Yan Zhang, Qingsong Hu, Jiayuan Wu, Haigang Ren, Chun-Feng Liu, Guanghui Wang

**Affiliations:** 1 Department of Pharmacology, Laboratory of Molecular Neuropathology, Jiangsu Key laboratory of Translational Research and Therapy for Neuropsychiatric disorders, College of Pharmaceutical Sciences, Soochow University, 199 Ren’ai Road, Suzhou 215123, Jiangsu, China; 2 Department of Neurology and Suzhou Clinical Research Center of Neurological Disease, The Second Affiliated Hospital of Soochow University, Suzhou 215004, China

## Abstract

Parkinson’s disease (PD) is the second most prevalent neurodegenerative disease. Although its pathogenesis remains unclear, mitochondrial dysfunction plays a vital role in the pathology of PD. P7C3, an aminopropyl carbazole, possesses a significant neuroprotective ability in several neurodegenerative disorders, including PD. Here, we showed that P7C3 stabilized mitochondrial membrane potential, reduced reactive oxygen species production, and inhibited cytochrome *c* release in MES23.5 cells (a dopaminergic (DA) cell line) exposed to 1-methyl-4-phenylpyridinium (MPP^+^). In MES23.5 cells, P7C3 inhibited glycogen synthase kinase-3 beta (GSK3*β*) activation induced by MPP^+^. P7C3 also inhibited p53 activity and repressed Bax upregulation to protect cells from MPP^+^ toxicity. In addition, the activation of p53 was significantly attenuated with the inhibition of GSK3*β* activity by P7C3. Furthermore, P7C3 blocked GSK3*β* and p53 activation in the midbrain, and prevented DA neuronal loss in the substantia nigra in 1-methyl-4-phenyl-1,2,3,4-tetrahydropyridine mice. Thus, our study demonstrates that P7C3 protects DA neurons from neurotoxin-induced cell death by repressing the GSK3*β*-p53-Bax pathway both *in vitro* and *in vivo*, thus providing a theoretical basis for P7C3 in the potential clinical treatment of PD.

P7C3 is a small aminopropyl carbazole compound that was first identified to have a remarkable neuroprotective ability for newborn hippocampal neurons from apoptosis.^1^ Thereafter, P7C3 was demonstrated to possess neuroprotective efficacy in many diseases or brain injury. P7C3 and its variant P7C3A20 exhibit neuroprotective efficacy in traumatic brain injury^2, 3^ and stroke.^4, 5^ This compound can also induce postnatal hippocampal neurogenesis, showing antidepressant efficacy in mice^6, 7^ and improve the scopolamine-induced learning and memory impairment.^8^ Importantly, P7C3 and its variants exhibit strong neuroprotective efficacy in many neurodegenerative disease models. In the G93A-SOD1 mutant mouse model of amyotrophic lateral sclerosis, the early administration of P7C3 and its variant P7C3A20 at or before disease onset effectively blocks motor neuronal death.^9^ P7C3 also significantly relieves the tyrosine hydroxylase (TH) neuronal loss in the substantia nigra (SN) and striatum in 1-methyl-4-phenyl-1,2,3,4-tetrahydropyridine (MPTP) mouse or 6-hydroxydopamine (6-OHDA) rat model of Parkinson’s disease (PD).^10, 11^ Recently, P7C3 and its variants were shown to bind to and enhance the activity of nicotinamide phosphoribosyltransferase, thereby increasing nicotinamide adenine dinucleotide levels to protect cells.^12^

PD is one of the most common neurodegenerative disorders, following only Alzheimer’s disease (AD).^13^ The preferential loss of dopaminergic (DA) neurons in the SN leads to dopamine depletion in the striatum, resulting in symptoms of bradykinesia, resting tremor, muscle rigidity, and flexed posture.^14^ Although the pathogenesis of PD remains obscure, evidence of the etiology of PD is increasing. For example, oxidative stress^15^ and mitochondrial dysfunction^16^ are considered significant elements of DA neuronal death in PD. Mitochondrial dysfunction is an important factor associated with PD pathogenesis. In the SN of PD brains, mitochondrial complex I activity is significantly decreased,^17^ thus providing a link between mitochondrial dysfunction and PD.

The direct link between mitochondrial dysfunction and PD was demonstrated by evidence suggesting that MPTP induces PD symptoms in drug-abuse patients.^18^ MPTP, a special neurotoxin that can selectively damage DA neurons, is widely applied to establish PD animal models.^19^ When MPTP penetrates the blood–brain barrier, it can be metabolized into 1-methyl-4-phenylpyridinium (MPP^+^) by monoamine oxidase in glial cells; MPP^+^ is then taken up into DA neurons through the dopamine transporter.^20^ MPP^+^ inhibits mitochondrial complex I to interfere with the electron transport chain, resulting in ATP depletion and reactive oxygen species (ROS) production.^21^ ROS induces mitochondrial damage, leading to the loss of membrane potential and release of cytochrome *c* (Cyt *c*) from mitochondria, which activates caspase-3 and induces apoptosis.^22^

Although P7C3 exerts protective effects in animal models of PD, the mechanisms underlying its protection in PD remain unclear. As P7C3 has multiple protective effects in many different neuronal diseases, it is likely that this compound may function in different pathways in different diseases, including PD. In this study, we showed that P7C3 suppresses the activity of glycogen synthase kinase-3 beta (GSK3*β*) induced by MPP^+^ and MPTP, leading to the inhibition of p53 activation and Bax expression, thus impeding mitochondrial damage and eventually protecting DA neurons from death in MPTP mouse models of PD.

## Results

### P7C3 attenuates MPP^+^-induced cytotoxicity in MES23.5 cells

**Figure 1:**
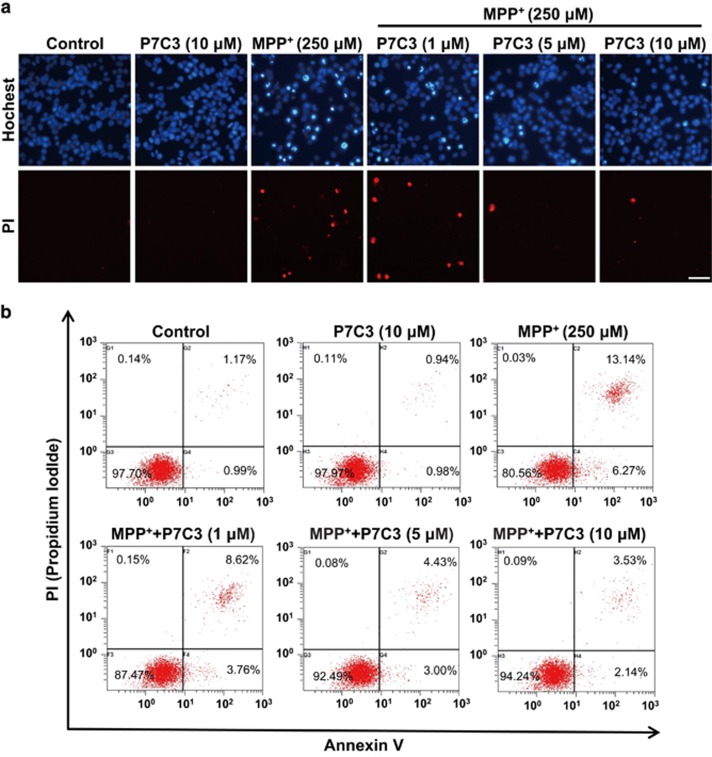
P7C3 attenuates MPP^+^ cytotoxicity in MES23.5 cells. (**a**) MES23.5 cells were pretreated with P7C3 (1, 5, or 10 *μ*M) for 2 h and then exposed to MPP^+^ (250 *μ*M) for 24 h. After treatment, the cells were subjected to Hoechst or PI staining to detect cell death. Scale bar, 10 *μ*m (**b**) MES23.5 cells were treated as in **a** and subjected to flow cytometry analysis

MPP^+^ damages DA neurons through the inhibition of complex I of the mitochondrial respiratory chain.^22^ We therefore detected the protective effects of P7C3 on MPP^+^-induced DA neuronal death *in vitro*. We observed that the pretreatment of P7C3 markedly attenuated the MPP^+^-induced cytotoxicity to MES23.5 cells, as evidenced by the Hochest 33342 and propidium iodide (PI) staining (Figures 1a and b). P7C3 strikingly decreased the number of the cells with condensed nuclei in response to treatment with MPP^+^ (Figure 1a). Moreover, the amount of MES23.5 cell death induced by MPP^+^ was also markedly reduced in the presence of P7C3 (Figure 1b).

### P7C3 alleviates MPP^+^-induced oxidative stress and mitochondrial damage in MES23.5 cells

**Figure 2:**
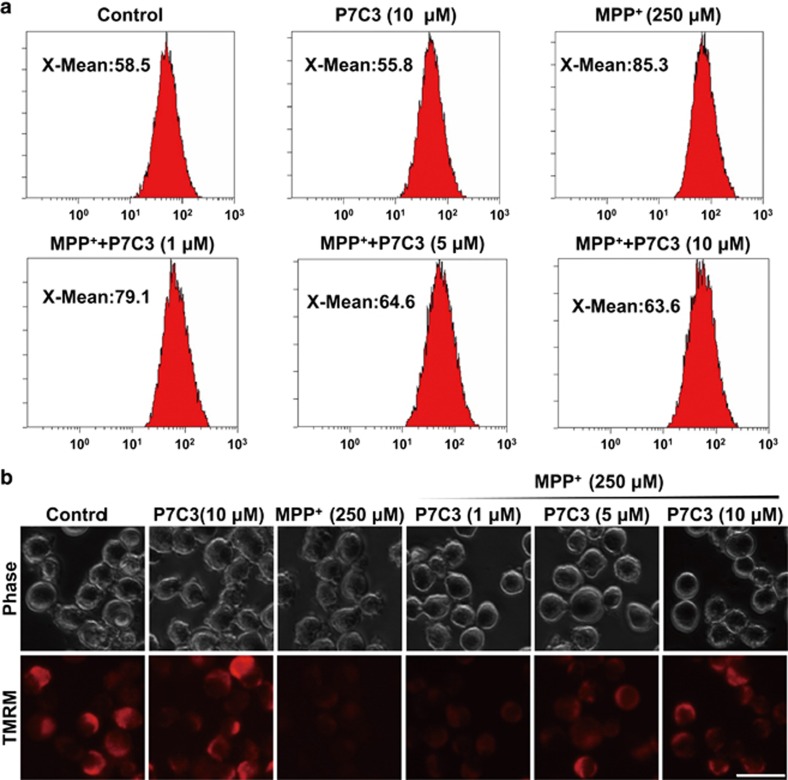
P7C3 alleviates MPP^+^-induced oxidative stress and mitochondrial damage in MES23.5. (**a**) MES23.5 cells were treated with various concentrations of P7C3 (1, 5, or 10 *μ*M) for 2 h followed by the treatment with MPP^+^ (250 *μ*M) for 24 h. The cells were then stained with a DCFH-DA probe to detect ROS production in cells using flow cytometry analysis. (**b**) MES23.5 cells were treated as in (**a**) and stained with TMRM to show the mitochondrial membrane potential. Scale bar, 10 *μ*m

Since MPP^+^ impairs DA neurons by inhibiting the mitochondrial complex I, resulting in the overproduction of ROS and dysfunction of mitochondria, including the loss of the mitochondrial membrane potential (MMP),^23^ we examined whether P7C3 affects ROS generation and stabilizes mitochondrial membrane to protect DA neurons against MPP^+^ toxicity. In MES23.5 cells, MPP^+^ largely increased ROS production, whereas pretreatment with P7C3 significantly decreased it (Figure 2a). Moreover, MPP^+^ largely decreased MMP, as indicated by tetramethylrhodamine methyl ester (TMRM), a dye sequestered by active mitochondria (Figure 2b). However, pretreatment with P7C3 significantly restored MMP in a dose-dependent manner (Figure 2b). P7C3, at a concentration of 10 *μ*M, almost completely restored the MMP impaired by MPP^+^ (Figure 2b). Moreover, P7C3 itself did not affect MMP (Figure 2b). These data suggest that P7C3 stabilizes MMP and inhibits ROS production to protect MES23.5 cells exposed to MPP^+^.

### P7C3 inhibits cytochrome *c* release and caspase-3 activation induced by MPP^+^

**Figure 3:**
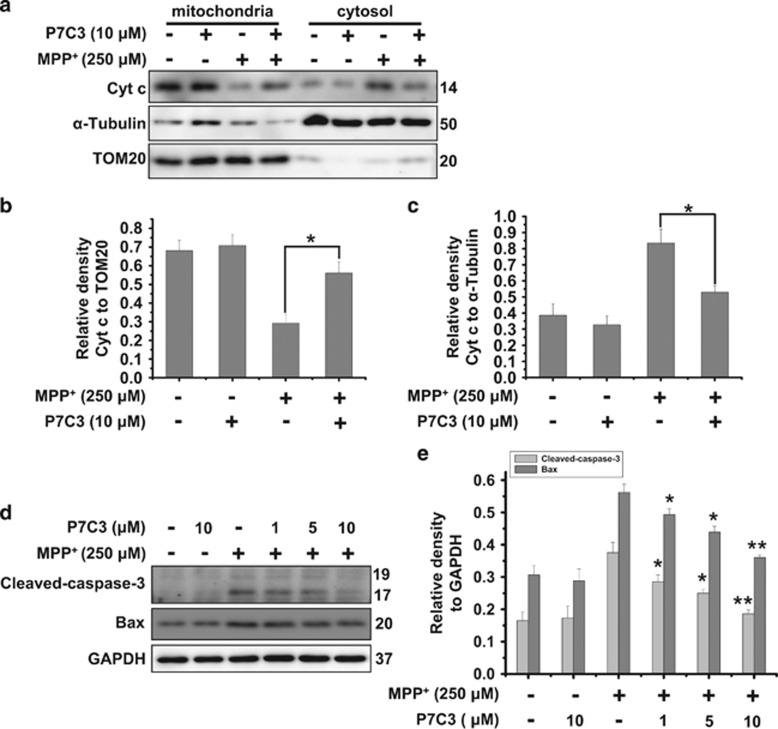
P7C3 inhibits MPP^+^-induced cytochrome *c* release and caspase-3 activation. (**a**) MES23.5 cells were treated with P7C3 (10 *μ*M) for 2 h and then incubated with MPP^+^ (250 *μ*M) for 24 h. After incubation, the mitochondrial and cytosolic fractions were separated using a Mitochondria Isolation Kit. The Cyt *c* protein levels in the mitochondria and cytosol were detected using immunoblot analysis. (**b** and **c**) The quantitative analyses of the relative density of Cyt *c* compared with loading controls in mitochondria (TOM20) or in the cytosol (*α*-Tubulin) are shown. The values are presented as the means ± S.E.M. from three independent experiments. *P<0.05, one-way ANOVA. (**d**) The MES23.5 cells were treated as in **a**. The cleaved caspase-3 and Bax protein levels were detected using immunoblot analysis. (**e**) The quantitative analysis of the relative density of cleaved caspase-3 and Bax compared with the loading control (GAPDH) is shown. The values are presented as the means ± S.E.M. from three independent experiments. *P<0.05, **P<0.01 *versus* the group treated with MPP^+^ alone using one-way ANOVA

The mitochondria play a significant role in apoptosis. Mitochondrial damage results in the release of Cyt *c*, which triggers caspases activation, and eventually induces apoptosis.^24^ To further confirm the effects of P7C3 on mitochondria, we performed mitochondrial fractionation assays to examine the release of Cyt *c* after MPP^+^ treatment in the presence or absence of P7C3 in MES23.5 cells. P7C3 strikingly blocked MPP^+^-induced Cyt *c* release from mitochondria to the cytosol (Figures 3a–c). The inhibition of caspase-3 cleavage by P7C3 was observed in cells treated with MPP^+^ (Figure 3d). The pro-apoptotic protein Bax, which is a Bcl-2 family protein, regulates and promotes permeability transition pore (PTP) opening.^25^ Thus, we examined Bax protein levels in cells treated with MPP^+^ in the presence of P7C3. Interestingly, P7C3 significantly decreased the Bax expression induced by MPP^+^ (Figure 3d). These data indicate a role for P7C3 in regulating the mitochondrial apoptotic pathway in cells exposed to MPP^+^.

### P7C3 suppresses mitochondria apoptosis by inhibiting of GSK3*β*

**Figure 4:**
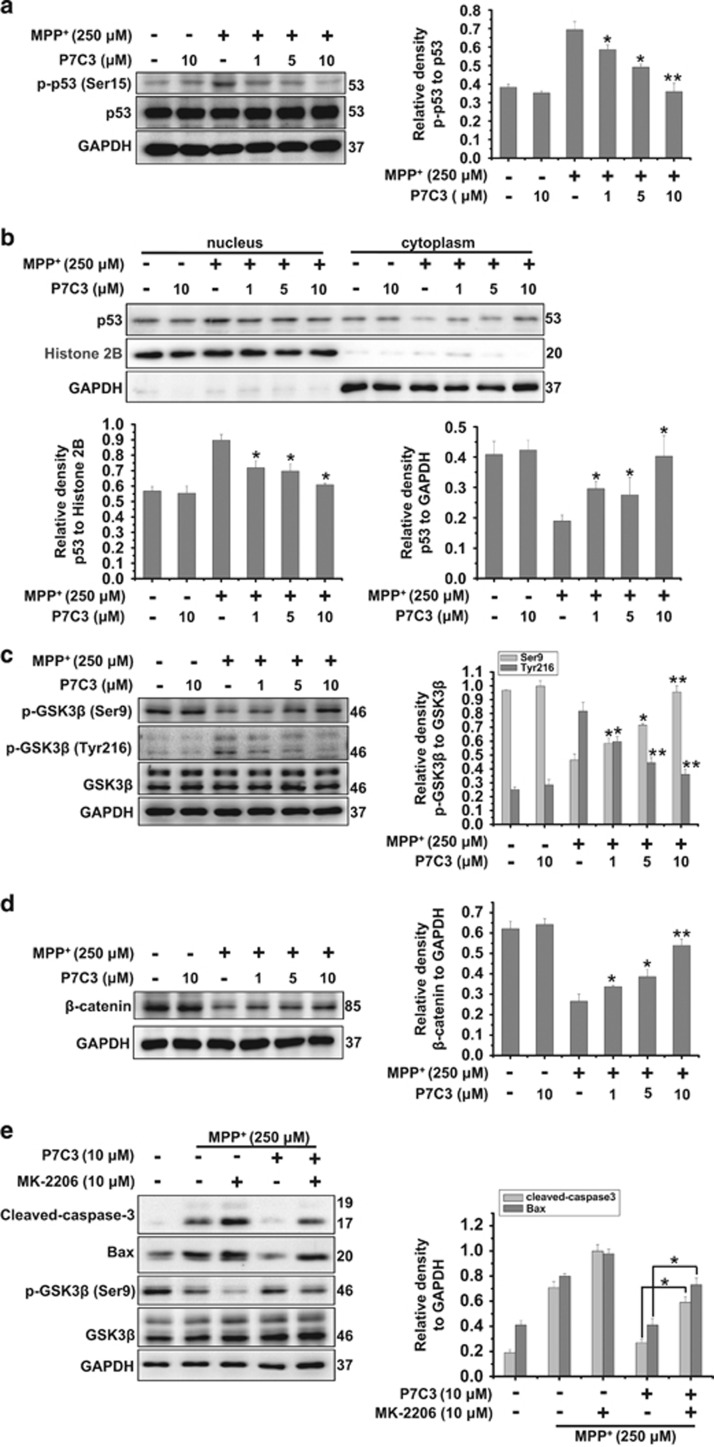
P7C3 suppresses p53 and GSK3*β* activations. (**a**) The MES23.5 cells were administrated at various concentrations of P7C3 (1, 5, or 10 *μ*M) for 2 h followed by treatment with MPP^+^ (250 *μ*M) for 24 h. The protein levels of p-p53 and p53 were measured using immunoblot analysis. The quantification of the intensity of p-p53 relative to p53 is shown in the right panel. *P<0.05, **P<0.01 *versus* the MPP+ alone group by one-way ANOVA. (**b**) The MES23.5 cells were treated as in **a**, followed by a preparation of nuclear and cytoplasmic fractions. The p53 protein levels in nucleus and cytoplasm were detected using immunoblot analysis. The quantitative analyses of the relative density of p53 to the loading controls in nucleus (Histone 2B) or in the cytoplasm (GAPDH) are shown in the lower panel. The values are presented as means ± S.E.M. from three independent experiments. *P<0.05 *versus* the group treated with MPP^+^ alone using one-way ANOVA. (**c**) The MES23.5 cells were treated as in (**a**). The protein levels of p-GSK3*β* (Ser9) and p-GSK3*β* (Tyr216) were detected using immunoblot analysis. The right panel displays the relative densities of these proteins relative to GSK3*β*. The values are presented as the means ± S.E.M. from three independent experiments. *P<0.05, **P<0.01 *versus* the group treated with MPP^+^ alone using one-way ANOVA. (**d**) The MES23.5 cells were treated as in (**a**). The protein levels of *β*-catenin were detected using immunoblot analyses. The right panel displays the relative density of *β*-catenin relative to GAPDH. The values are presented as the means ± S.E.M. from three independent experiments. *P<0.05, **P<0.01 *versus* the group treated with MPP^+^ alone using one-way ANOVA. (**e**) The MES23.5 cells were pretreated with P7C3 (10 *μ*M) in the presence or absence of MK-2206 (10 *μ*M) for 2 h, and then exposed to MPP^+^ (250 *μ*M) for 24 h. The protein levels of cleaved caspase-3, Bax, p-GSK3*β* (Ser9), GSK3*β* and GAPDH were measured using immunoblot analyses. The quantitative analyses of the relative density of cleaved caspase-3 and Bax compared with the loading control (GAPDH) are shown in the right panel. The values are presented as the means ± S.E.M. from three independent experiments. *P<0.05, one-way ANOVA

Because P7C3 inhibits MPP^+^-induced Bax expression (Figure 3d) and Bax is primarily transactivated by the transcription factor p53,^26^ we examined whether P7C3 influences p53. In MES23.5 cells treated with MPP^+^, pretreatment with P7C3 strikingly restrained p53 phosphorylation at serine 15 (Ser15) but not influenced its abundance (Figure 4a). In addition, the nuclear fractionation experiments showed that P7C3 prevented p53 nuclear translocation in MPP^+^-treated MES23.5 cells (Figure 4b), indicating that P7C3 inhibited the increase of p53 activity induced by MPP^+^. It has been reported that mitochondrial oxidative stress activates GSK3*β*^27^ and that an inhibition of GSK3*β* attenuates p53 activity.^28^ We therefore examined the effects of P7C3 on GSK3*β* activity by evaluating its phosphorylation at sites Ser9 (a site inhibiting GSK3*β* enzymatic activity)^29^ and Tyr216 (a site activating GSK3*β* enzymatic activity).^30^ In MES23.5 cells that were treated with MPP^+^, the phosphorylation of Ser9 in GSK3*β* was decreased (Figure 4c). However, that of tyrosine 216 was increased (Figure 4c), suggesting that MPP^+^ activates GSK3*β*. However, pretreatment with P7C3 significantly inhibited GSK3*β* activation mediated by MPP^+^ (Figure 4c). Moreover, the level of *β*-catenin, an important substrate of GSK3*β*,^31^ was increased upon P7C3 treatment in MPP^+^-treated cells (Figure 4d), suggesting an inhibition of GSK3*β* activity by P7C3. To further identify the inhibiting effects of P7C3 on GSK3*β* activation induced by MPP^+^, the specific inhibitor of AKT MK-2206 was introduced, which leads to an inhibition of the phosphorylation of GSK3*β* at Ser9 by AKT.^32^ MK-2206 treatment significantly repressed GSK3*β* phosphorylation at Ser9 and increased caspase-3 cleavage and Bax levels in MPP^+^-treated cells (Figure 4e). However, upon P7C3 treatment, the phosphorylation of GSK3*β* was partially restored in MPP^+^-treated cells that were treated with MK-2206 (Figure 4e).

### P7C3 possesses cytoprotective effects analogous to the GSK3*β* inhibitor

**Figure 5:**
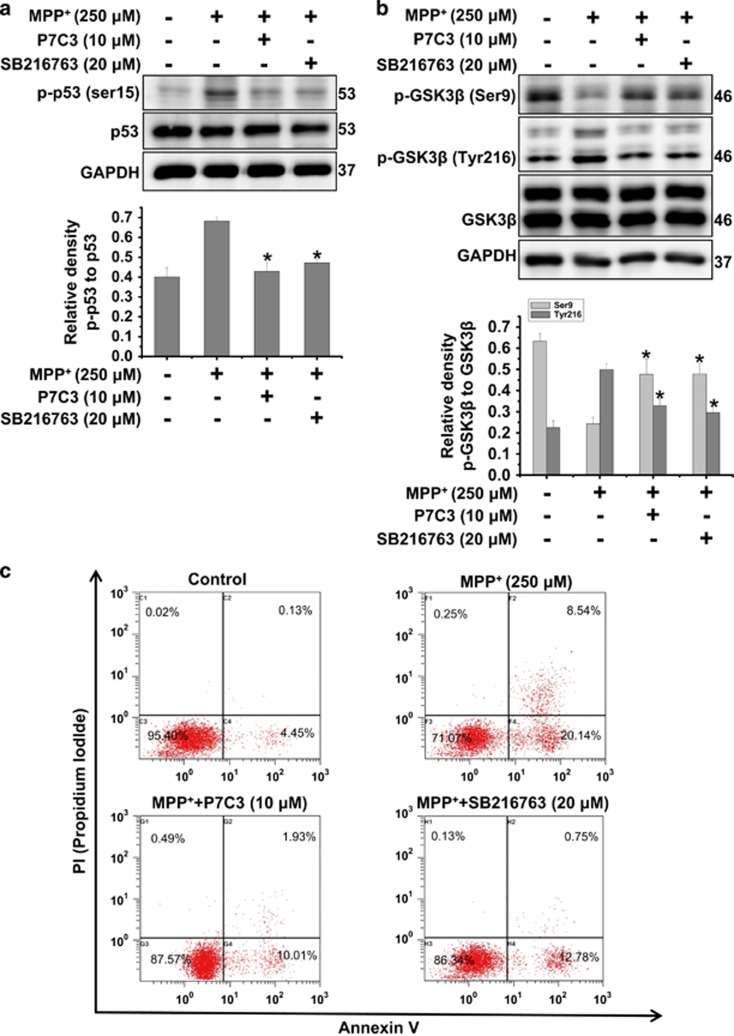
GSK3*β* inhibitor possesses the analogous cytoprotective effects compared with P7C3. (**a**) The MES23.5 cells were treated with P7C3 (10 *μ*M) or SB216763 (20 *μ*M) for 2 h, and then incubated with MPP^+^ (250 *μ*M) for 24 h. The p-p53, p53 protein levels were detected with immunoblot analyses. The bottom panel shows the band intensity of p-p53 relative to p53. (**b**) The MES23.5 cells were treated as in **a**. The protein levels of p-GSK3*β* (Ser9) and p-GSK3*β* (Tyr216) were measured using immunoblot analysis. The bottom panel displayed relative density of the proteins relative to GSK3*β*. The values are presented as the means ± S.E.M. from three independent experiments. *P<0.05 *versus* the MPP^+^ alone group using one-way ANOVA. (**c**) The MES23.5 cells were treated as in (**a**). The quantification of cell death measured using flow cytometry analysis is shown

To further identify that the inhibition of GSK3*β* by P7C3 plays a role in p53 activation, we compared the effects of P7C3 on p53 with those of the GSK3*β* inhibitor SB216763 in MPP^+^-treated MES23.5 cells. P7C3, similar to the GSK3*β* inhibitor SB216763, inhibited p53 phosphorylation (Figure 5a) and GSK3*β* activation (Figure 5b). In addition, P7C3 and SB216763 suppressed cell death in MPP^+^-treated MES23.5 cells, detected using flow cytometry (Figure 5c). Thus, these data suggest that the suppression of GSK3*β* activation by P7C3 inhibits p53 activation and protects cells against MPP^+^ toxicity.

### P7C3 inhibits GSK3*β* activity and protects MPTP-induced DA neuronal loss *in vivo*

**Figure 6:**
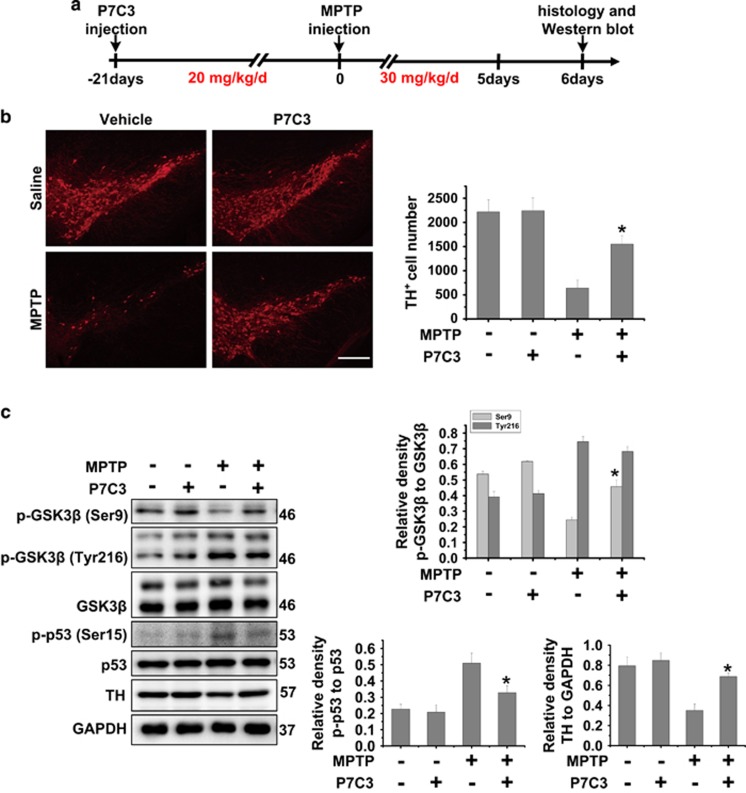
P7C3 inhibits GSK3*β* activity to protect DA neurons from MPTP toxicity *in vivo.* (**a**) A schematic diagram of animal experimental procedure is shown. (**b**) Twenty-four hours after the last intraperitoneal injection, mice were killed and fixed with perfusion. The brains were post-fixed with a fixing agent overnight at 4 °C, followed by the treatment with 30% sucrose at 4 °C for another night. Serial 20 *μ*M-thick mouse midbrain slices were cut using a freezing microtome. Immunohistochemical staining was conducted using anti-TH antibody. The quantification of TH^+^ cell numbers is shown in the right panel. Scale bar, 10 *μ*m. *n*=4 per group. (**c**) Twenty-four hours after the last intraperitoneal injection, the fresh midbrains samples were collected and subjected to immunoblot analysis with indicated antibodies. *n*=4 per group. The protein levels of p-GSK3*β* (Ser9), p-GSK3*β* (Tyr216), GSK3*β*, p53, p-p53, TH and GAPDH were measured using immunoblot analysis. The quantification of the intensity of p-GSK3*β* (Ser9) and p-GSK3*β* (Tyr216) relative to GSK3*β*, p-p53 relative to p53, and TH relative to GAPDH are shown in the right panels. *n*=4 per group. The values are presented as means ± S.E.M. from three independent experiments. *P<0.05 *versus* the group treated with MPTP alone using one-way ANOVA

To further identify the role of P7C3, we utilized a MPTP mouse model to examine the effects of P7C3 on GSK3*β* and neuroprotection. The animals were subjected to continuous P7C3 intraperitoneal injection for 21 days, followed by the intraperitoneal injection of MPTP for 5 days (Figure 6a). Pretreatment with P7C3 significantly blocked MPTP-induced TH neuronal loss in the SN (Figure 6b). In addition, P7C3 inhibited MPTP-induced GSK3*β* and p53 activation in the midbrain (Figure 6c). Consistent with the immunohistochemical data (Figure 6b), P7C3 recovered the TH protein levels in MPTP mice (Figure 6c).

## Discussion

**Figure 7:**
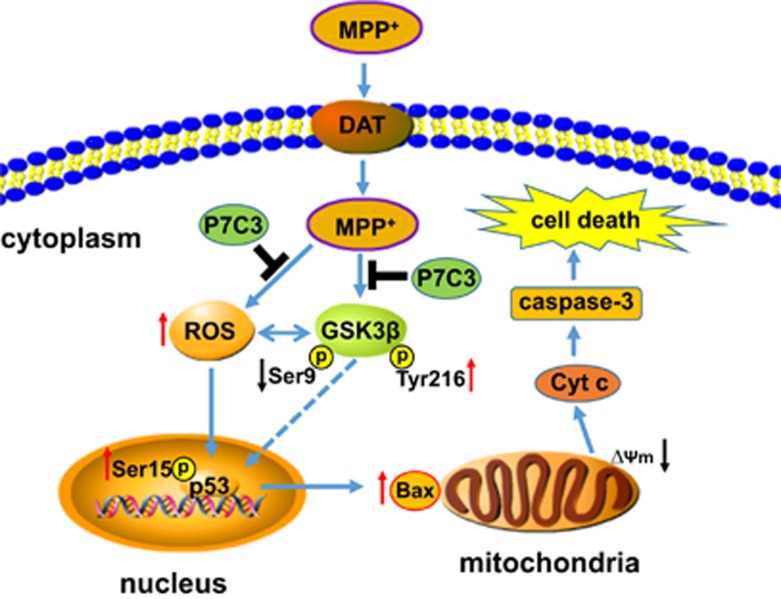
A schematic diagram showing the involvement of the GSK3*β*/p53/Bax pathway by which P7C3 protects DA neurons from MPP^+^-induced neurotoxicity. Once MPP^+^ is taken into DA neurons through dopamine transporter (DAT), this molecule both induces ROS production due to mitochondrial complex I inhibition and activates GSK3*β* indicated by the decrease of phosphorylation at Ser9 (a site inhibiting GSK3*β* enzymatic activity) and the increase of phosphorylation at Tyr216 (a site activating GSK3*β* enzymatic activity). On one hand, ROS activates GSK3*β*, on the other hand, GSK3*β* also increases ROS generation in turn. Subsequently, ROS overproduction and GSK3*β* activation activate p53, which transactivates Bax expression, leading to the opening of the mitochondrial PTP and the release of Cyt *c*, which activates caspase-3 and induces cell death. However, pretreatment with P7C3 inhibits GSK3*β* activation. Thus, the suppression of p53 activation and Bax expression protects mitochondria from damage and eventually attenuates MPP^+^-induced neurotoxicity

It has been reported that P7C3 possesses the neuroprotective efficacy in neurodegenerative disease animal models.^9, 10, 11^ In PD animal models, P7C3 protects DA neurons from MPTP and 6-OHDA toxicity.^10, 11^ However, it is still unknown in which pathway P7C3 exerts protective effects in PD models. Here, the present study showed that P7C3 functions in the GSK3*β*/p53/Bax pathway to protect mitochondria from MPP^+^- or MPTP-induced damage, thereby improving the survival of DA neurons *in vitro* and *in vivo* (Figure 7).

GSK3*β* is associated with various neurodegenerative diseases including AD and PD.^33^ GSK3*β* activation is involved in both neurotoxin-induced and genetic factor-induced DA neuronal loss.^34^ Accordingly, the active form of GSK3*β* (p-GSK3*β* Tyr216) is increased in the striatum of postmortem samples of PD.^35^ In addition, MPTP animals show the hyperphosphorylation of Tyr216 and dephosphorylation of Ser9 in GSK3*β*.^36^ The inhibition of GSK3*β* protects DA neurons from MPTP toxicity.^37^ In our observations, P7C3 protects DA neurons from MPP^+^- and MPTP-induced cell death, showing a significant protective efficacy *in vitro* and *in vivo*. However, in the MPP^+^ cellular model, P7C3 inhibited MPP^+^-induced GSK3*β* activation, indicated as repressed phosphorylation at Tyr216 and increased phosphorylation at Ser9. In MPTP mice, P7C3 markedly blocked the dephosphorylation of GSK3*β* at Ser9, without an obvious influence on Tyr216. The slightly different effects of P7C3 on MPTP-mediated GSK3*β* activation between *in vitro* and *in vivo* suggest that some factors may be differentially involved in the phosphorylation of Tyr216 in GSK3*β*, and P7C3 has a preferential effect on Ser9 in modulating GSK3*β* activation *in vivo*.

Damage to mitochondria by MPP^+^ or MPTP is caused by an induction of ROS production due to an inhibition of mitochondrial complex I, which results in mitochondrial oxidative stress.^21^ It has been reported that mitochondrial oxidative stress activates GSK3*β* and Bax, leading to mitochondrial PTP opening.^27^ The opening of PTP triggers a feed-forward loop involving a progressive Ca^2+^ surge, which further induces ROS production and PTP opening.^38^ The inhibition of GSK3*β* blocks oxidative stress-induced PTP opening.^27, 39^ Most interestingly, GSK3*β* activation occurs in a ROS-dependent manner, and in turn induces PTP opening.^27^ However, GSK3*β* inhibition also prevents ROS production.^40^ As GSK3*β* inhibitor SB216763 also decreases its activation as P7C3 in MPP^+^-treated cells, it seems that ROS and GSK3*β* influence each other. In our observations, P7C3 inhibited MPP^+^-induced GSK3*β* activation and Bax upregulation to stabilize the mitochondrial membrane and block Cyt *c* release, suggesting a role for P7C3 in mitochondrial protection by the abrogation of PTP sensitization via the inhibition of GSK3*β*.

The opening of PTP results from the oligomerization of Bax or Bak on the mitochondrial membrane, leading to a loss of MMP and release of mitochondrial components.^41^ The release of Cyt *c* activates caspases to induce cell death.^41^ Bax is a pro-apoptotic protein that is transactivated by the transcription factor p53.^26^ GSK3*β* acts upstream of caspases and Cyt *c* release.^42, 43^ Most importantly, GSK3*β* interacts with and promotes the transcriptional activity of p53.^28, 42^ The dominant-negative mutant of p53 blocks GSK3*β*-induced apoptosis.^44^ suggesting that GSK3*β* influences p53, contributing to apoptosis. The phosphorylation of Ser15 plays a critical role in activating p53 upon stimulation.^45^ Oxidative stress induces p53 phosphorylation at Ser15, but not serine 9, 20, or 392.^46^ In addition, oxidative stress does not influence p53 protein levels.^46^ Although there is no direct evidence to prove that GSK3*β* activation directly promotes p53 phosphorylation at Ser15, GSK3*β* inhibition reduces ROS generation.^40, 47^ It is possible that the inhibition of p53 phosphorylation by P7C3 may result from the inhibition of ROS generation that is associated with GSK3*β* activation. In our study, P7C3 and GSK3*β* inhibitor SB216763 effectively blocked the MPP^+^-induced phosphorylation of Ser15 in p53, thus repressing DA neuronal death. In addition, P7C3 blocked MPTP-induced GSK3*β* and p53 activation to promote the survival of DA neurons in the SN *in vivo*.

In summary, we showed that P7C3 inhibits GSK3*β* activation to protect DA neurons from MPP^+^- or MPTP-induced toxicity *in vitro* or *in vivo*. Thus, our study provides a mechanistic explanation for the neuroprotective efficacy of P7C3 on surviving DA neurons in MPP^+^ cellular and MPTP animal models of PD.

## Materials and Methods

### Animal experiments

Male C57BL/6 mice, 25–30 g, were purchased from SLACCAL Lab Animal Ltd (Shanghai, China). The mice were housed under conditions at 20–26 °C, 50–60% relative humidity and a 12-h light and a 12-h dark cycle. The animals were provided free access to food and water. For total protein acquisition in the SN, mice were killed after the last drug treatment. The midbrains were obtained and homogenized in lysis buffer for immunoblot analysis. All experiments were conducted according to the Regulations of Experimental Animal Administration issued by the Animal Committee of Soochow University. For drug treatment *in vivo*, the mice were randomly divided into four groups: (1) vehicle + saline group; (2) P7C3 + saline group; (3) vehicle + MPTP group; and (4) P7C3 + MPTP group. Each mouse was administrated twice daily with vehicle or P7C3 at a dose of 20 mg/kg per day for 21 consecutive days through intraperitoneal injection. From the day 22, mice in groups (3) and (4) were intraperitoneally administered MPTP at a dose of 30 mg/kg per day for 5 days. The mice in groups (1) and (2) were treated with the same volume of saline.

### Cell culture and drug treatment

MES23.5 cells, a DA cell line, were kindly gifted from Dr. Wei-Dong Le (Baylor College of Medicine, Houston, TX, USA), and cultured in DMEM/F12 supplemented with Sato's components containing 5% heat-inactivated fetal bovine serum including penicillin (100 U/ml) and streptomycin (100 *μ*g/ml). P7C3 was purchased from Hanxiang Technology (Shanghai, China) and dissolved in dimethylsulfoxide (DMSO). MPP^+^ and MPTP were purchased from Sigma (St. Louis, MO, USA) and dissolved with PBS and saline, respectively. MES23.5 cells were pretreated with a gradient concentration of P7C3, and then exposed to MPP^+^ for 24 h, followed by the detection of apoptosis

### Hochest and propidium iodide staining assays

MES23.5 cells were treated with MPP^+^ with or without pretreatment with varying concentrations of P7C3. Then, the cells were incubated with Hoechst 33342 (Sigma) or PI (Beyotime, Shanghai, China) for 5 min and washed with PBS. The cells were observed using an inverted IX71 microscope system (Olympus, Tokyo, Japan).

### Subcellular fractionation assay

MES23.5 cells were treated with P7C3 for 2 h. After washing, the cells were treated with MPP^+^ for 24 h. The cells were then collected, and cytosolic and mitochondrial fractions were isolated using the Mitochondria Isolation Kit for cultured cells (Beyotime). For immunoblot analysis, TOM20 (Santa Cruz Biotechnology, Santa Cruz, CA, USA) and *α*-Tubulin (Abcam, Cambridge, UK) were selected as the markers for mitochondria and cytosol, respectively. For obtaining the nuclear and cytoplasmic fractions, cells were treated as described above and then lysed in the fractionation buffer containing 3 mM CaCl_2_, 2 mM MgAc, 320 mM sucrose, 0.1 mM EDTA, 1 mM DTT, 0.5 mM phenylmethylsulfonyl fluoride (PMSF), and 0.5% NP-40 for 20 min on ice. After centrifugation for 15 min at 600 × *g* at 4 °C, the supernatant was collected as the cytoplasmic fraction. The pellets were washed twice with the fractionation buffer without NP-40 and lysed in the nuclear lysis buffer containing 280 mM KCl, 0.2 mM EDTA, 1 mM DTT, 0.5 mM PMSF, 20 mM Hepes (pH 7.9), 25% glycerol, 1.5 mM MgCl_2_, and 0.3% NP-40 as the nuclear fraction. Histone 2B (Abcam) and anti-glyceraldehyde-3-phosphate dehydrogenase (GAPDH) (Millipore, Billerica, MA, USA) were selected, respectively, as the markers for nucleus and cytoplasm in immunoblot analyses.

### Mitochondrial membrane potential measurement

To measure the MMP, P7C3- or MPP^+^-treated MES23.5 cells were incubated with 100 nmol/l TMRM (Sigma) in PBS for 15 min at 37 °C. Healthy mitochondria were indicated with TMRM in red, while damaged mitochondria rapidly lost the red fluorescence, implying the loss of MMP. After incubation, MES23.5 cells were immediately washed with PBS and then resuspended in PBS. Finally, the cells were observed using an inverted IX71 microscope system (Olympus).

### Measurement of reactive oxygen species production

P7C3- or MPP^+^-treated MES23.5 cells were incubated in serum-free media with 5 *μ*M 2’,7’-dichlorofluorescin diacetate (DCFH-DA) (Beyotime) at 37 °C for 20 min. DCFH-DA transforms into fluorescent DCF to assess the generation of intracellular ROS. The cells were then washed with PBS and resuspended in PBS. Intracellular ROS were measured using flow cytometry.

### Quantification detection of apoptosis

MES23.5 cells were administrated as described above. Apoptosis was measured by flow cytometry using the Annexin V-FITC Apoptosis Detection Kit (Beyotime) according to the manufacturer’s instructions.

### Immunoblot analysis and antibodies

MES23.5 cells were lysed in 1 × SDS lysis buffer (150 mmol/l NaCl, 25 mmol/l Tris-HCl, pH 7.6, 1% sodium deoxycholate, and 1% NP-40) with a protease inhibitor cocktail (Roche, Basel, Switzerland). Nearly 20 *μ*g of cell lysate was isolated by SDS-PAGE and then transferred onto a PVDF membrane (Millipore). Immunoblot analysis was administrated with the following primary antibodies: anti-p-glycogen synthase kinase-3 beta (GSK3*β*) (Ser9), anti-p-GSK3*β* (Tyr216), anti-Cyt *c*, anti-*α*-Tubulin antibodies (Abcam); anti-p-p53 (Ser15) antibodies (Cell Signaling Technology, Danvers, MA, USA); anti-Bax, anti-p53, anti-GSK3*α*/*β*, anti-TOM20 antibodies (Santa Cruz Biotechnology); GAPDH, anti-TH antibodies (Millipore). The secondary antibodies, sheep anti-rabbit, or anti-mouse IgG-HRP were obtained from Thermo Fisher (Waltham, MA, USA). The proteins were visualized using an ECL detection kit (Thermo Fisher).

### Immunohistochemistry

Male C57BL/6 mice, 25–30 g, were administrated P7C3 or MPTP as described in the animal experiments above. Then, the mice were perfused with 0.9% saline, followed by 4% paraformaldehyde in 0.1 M PBS (pH 7.4). After perfusion, the mice brains were harvested for post-fixation in the same fixing agent overnight at 4 °C, followed by the treatment with the 30% sucrose at 4 °C for another night. Serial 20 *μ*M-thick mouse midbrain slices were prepared with a freezing microtome. Immunohistochemical staining was conducted using anti-TH antibodies (Millipore) against six slices per mouse (120 *μ*m interval). After incubation with primary antibody at room temperature for 6 h, the slices were incubated with rhodamine (red)- or FITC (green)-conjugated secondary antibody (Invitrogen, Carlsbad, CA, USA) for 2 h. Finally, the slices were stained with DAPI for 5 min and observed using an inverted IX71 microscope system (Olympus). TH^+^ neurons were counted with Image J software (National Institutes of Health, Bethesda, MD, USA) for each slice by two blinded investigators.

### Statistical analysis

The quantification analysis of immunoblots from three independent experiments was conducted using Photoshop 7.0 (Adobe, San Jose, CA, USA). The data were analyzed using origin 6.0 (Originlab, Northampton, MA, USA). Statistical significance was evaluated using one-way ANOVA. The criterion of significance was set at *P*<0.05. The values are shown as the means±S.E.M.
